# Global Warming: Clinton Climate Initiative Heats Up

**DOI:** 10.1289/ehp.114-a638b

**Published:** 2006-11

**Authors:** Tanya Tillett

Given that metropolitan areas account for more than 75% of the world’s greenhouse gas (GHG) emissions, it is only right that cities should lead the effort to stem such emissions. To this end, the William J. Clinton Foundation has partnered with the Large Cities Climate Leadership Group to launch the Clinton Climate Initiative (CCI). “The partnership . . . will take practical, and most importantly, measurable steps toward helping to slow down global warming, and by taking this approach I think we can make a big difference,” Clinton said at the CCI’s launch.

The first CCI project will create a purchasing consortium that will allow participating cities to save money on buying and developing energy-saving products and measures. Each member city will also use a web-based communication system and measurement tools created through the CCI to establish a baseline of its GHG emissions and report on progress as changes are implemented. Already, 24 of the world’s largest cities have pledged to support the voluntary effort, and many more have been invited to join.

Global warming experts applaud the CCI’s mitigation component, and see it as an integral first step in slowing the rate of global warming. Kristie Ebi, an independent consultant to UN agencies and others on climate and health issues, sees opportunity for the alliance to reduce GHG emissions in the next decade “by focusing on projects that increase energy efficiency, thus decreasing emissions from electric power generation.” She also points to transportation as a prime area for innovation, since this sector accounts for about one-third of all GHG emissions.

In the meantime, Ebi says, “Large cities also need to design and implement adaptation measures to reduce their [current] climate-related risks.” Some cities might implement heat wave early warning systems, for example, while others would benefit from water conservation measures.

John Reilly, associate director for research of the Massachusetts Institute of Technology Joint Program on the Science and Policy of Global Change, agrees: “Global warming is a result of the accumulation over many years of long-lived greenhouse gases. To have a tangible effect, an initiative must change emissions by a large amount and continue for decades.” Not even the CCI will noticeably affect temperature change within the next 50 years, Reilly says, but “the only way we will get to the year 2100 and look back and say we have made a substantial difference is to start out with firm steps toward a less GHG-intensive world than we otherwise are headed on.”

## Figures and Tables

**Figure:**
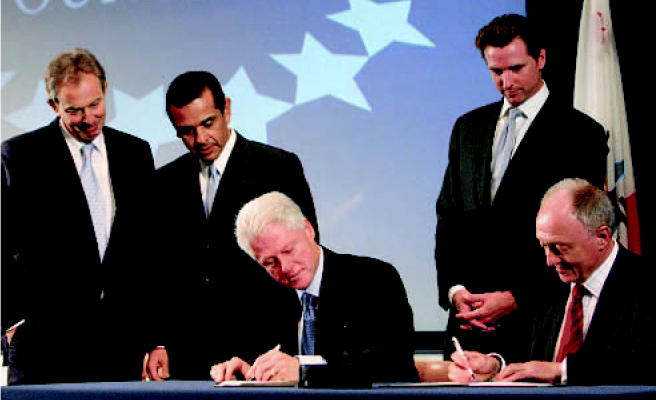
Men of action Former president Bill Clinton and London mayor Ken Livingstone (seated, left to right) sign the memorandum of understanding to launch the Clinton Climate Initiative. Joining them (standing, left to right) were British prime minister Tony Blair, Los Angeles mayor Antonio Villaraigosa, and San Francisco mayor Gavin Newsom.

